# Vitamin D receptor gene polymorphism predicts left ventricular hypertrophy in maintenance hemodialysis

**DOI:** 10.1186/s12882-021-02640-3

**Published:** 2022-01-15

**Authors:** Bingman Liu, Qingqing Yang, Liangyu Zhao, Hua Shui, Xiaoyun Si

**Affiliations:** Department of Nephrology, Zhongnan Hospital of Wuhan University, No.169 Donghu Road, Wuchang District, Wuhan, 430071 China

**Keywords:** Vitamin D receptor, Left ventricular hypertrophy, Bsm I, Maintenance hemodialysis

## Abstract

**Background:**

To verify that the single nucleotide polymorphisms (SNP) of vitamin D receptor (VDR) may lead to genetic susceptibility to left ventricular hypertrophy (LVH), the present study was designed to study four SNPs of VDR associated with LVH in maintenance hemodialysis (MHD) patients of Han nationality.

**Methods:**

120 MHD patients were recruited at Department of Nephrology, Zhongnan Hospital of Wuhan University to analyze the expression of genotype, allele and haplotype of Fok I, Bsm I, Apa I and Taq I in blood samples, and to explore their correlation with blood biochemical indexes and ventricular remodeling.

**Results:**

The results showed that the risks of CVD included gender, dialysis time, heart rate, SBP, glycated hemoglobin, calcium, iPTH and CRP concentration. Moreover, LAD, LVDd, LVDs, IVST and LVMI in B allele of Bsm I increased significantly. Fok I, Apa I and Taq I polymorphisms have no significant difference between MHD with LVH and without LVH. Further study showed that VDR expression level decreased significantly in MHD patients with LVH, and the B allele was positively correlated with VDR Expression.

**Conclusion:**

VDR Bsm I gene polymorphism may predict cardiovascular disease risk of MDH patients, and provided theoretical basis for early detection and prevention of cardiovascular complications.

## Introduction

With the continuous progress of renal replacement therapy, the survival rate and life quality of patients with end-stage renal disease have gradually improved, but at present, 50% of maintenance hemodialysis (MHD) patients die from cardiovascular disease (CVD), among which the incidence of left ventricular hypertrophy (LVH), coronary heart disease and heart failure are about 75%, 40% and 40%, respectively. A report shows that the mortality rate of CVD in young MHD patients is 500 times that in ordinary people of the same age, and that in elderly MHD patients is 5 times that in ordinary people of the same age [1]. Chronic renal failure has something in common with the cardiovascular risk factors of the general population, including diabetes and hypertension, but it also has its specific cardiovascular risk factors, such as LVH and left ventricular diastolic dysfunction, which are strong predictors of the mortality of MHD patients within two years [2]. In the past, it was thought that these two diseases were related to hypertension or cardiovascular calcification. Recent studies have found that these diseases also exist in patients with chronic renal failure under 20 years without cardiovascular calcification and hypertension. Traditional cardiovascular risk factors, such as age, dialysis age, systolic blood pressure, total cholesterol and low density lipoprotein, can not fully reflect the risk of cardiovascular disease in MHD patients. Searching for new CVD risk factors in MHD patients will help to evaluate the disease more accurately and improve the prognosis.

Vitamin D receptor (VDR) is a member of steroid hormone/thyroid hormone receptor superfamily, locating on the long arm of chromosome 12 and consists of 14 exons and multiple introns. The protein product contains 427 amino acids. Intracellular VDR combines with l,25-(OH)2D_3_ hormone signal molecule to form hormone-receptor complex, which combines with hormone response element on specific DNA sequences of target genes, thus regulating the expression of structural genes. Previous study showed that VDR knockout mice revealed active vitamin D as a promising agent inhibiting LVH progression [3]. However, a forty-eight week therapy with VDR receptor agonist paricalcitol did not alter left ventricular mass index or improve certain measures of diastolic dysfunction in patients with chronic kidney disease. Another trial showed that 52 weeks of treatment with oral paricalcitol (1 μg one time daily) significantly improved secondary hyperparathyroidism but did not alter measures of LV structure and function in patients with severe CK D[4]. Because of the inconsistency, it is speculated that the role of VDR gene polymorphism in CVD can not be ignored, and VDR gene polymorphism may become a new predictor of cardiovascular risk factors. Many single nucleotide polymorphisms (SNP) have been found in VDR gene, among which Fok I (rs222857), Bsm I (rs1544410), Apa I (rs7975232) and Taq I (rs731236), which are located at transcription initiation site, intron VII and exon IX, respectively. f, b, a, and t are used to represent polymorphic sites with these four endonucleases, and F, B, A, and T are used to represent polymorphic sites without these four endonucleases [5]. Teitcher [6] detected four alleles of VDR in 74 patients with Fabry disease characterized by LVH, arterial intimal calcification, progressive renal failure and neuropathy, and found that Taq I tt genotype has better cardiorenal protection than TT genotype, and the clinical symptoms are obviously alleviated. The genotype combination of T-A-f-B and t-a-F-b indicated severe organ damage and functional variation. Ortlepp [7] compared the expression of Bsm I allele between 100 patients with aortic stenosis and calcification and normal control group, and found that allele B was significantly correlated with aortic stenosis and calcification.

At present, the relationship between VDR gene polymorphism and LVH in MHD patients has been preliminarily studied. Testa [8] found that B allele was significantly correlated with the occurrence of LVH in Bsm I gene polymorphism sites of MHD patients, and left ventricular mass index (LVMI) increased significantly in BB genotype patients. It is speculated that the allele may lead to susceptibility to LVH by changing the structure and function of VDR. Are the four genetic polymorphisms of VDR associated with LVH in MHD patients of Han nationality? Is the correlation characterized by single SNP or synergistic effect of multiple SNPs? At present, there is no related report. Therefore, in this study, 120 MHD patients were chosen to analyze the expression of genotypes, allele and haplotype of Fok I, Bsm I, Apa I and Taq I in blood samples, and to explore their correlation with blood biochemical indexes and ventricular remodeling, so as to provide theoretical basis for early detection and active prevention of cardiovascular complications and improvement of prognosis of patients with end-stage renal disease.

## Materials and methods

### Participants

One hundred twenty maintenance hemodialysis patients, whose age, sex, body mass index (BMI) and general conditions matched were chosen from March 2018 to March 2019 at Department of Nephrology, Zhongnan Hospital of Wuhan University. We applied the on-line sample calculation formula (http://powerandsamplesize.com/Calculators/) to meet the sample requirements of randomized controlled trials. All patients were in relatively stable condition, without infection, inflammation, severe liver and other chronic diseases, and without obvious clinical manifestations of heart failure. No adverse cardiovascular events (including acute myocardial infarction, unstable angina pectoris, cerebrovascular diseases and peripheral vascular embolism) occurred within 3 months, and no major surgery was performed. The gender, age, dialysis time, systolic pressure and diastolic pressure of MHD patients were recorded. The patient's height and weight were measured and BMI were calculated: BMI = weight (kg )/ height (m^2^)

### Biochemical assays

Blood samples were obtained from all subjects every two months before dialysis. Blood samples were centrifuged at 1,500 *g* for 10 min at 4°C, and the plasma was immediately frozen and stored at -80°C. Serum immunoreactive parathyroid hormone (iPTH) was determined by Roche 2010 electrochemiluminescence method (Roche Diagnostics, Burgess Hill, UK). At the same time, serum calcium, phosphorus, total cholesterol, low density lipoprotein cholesterol, hemoglobin, serum albumin, serum creatinine and urea nitrogen were detected, and the urea clearance index (Kt/v) was calculated. And all the biochemical items were determined by Hitachi 7180. Take the average value within 6 months as the observation index.

### Color Doppler ultrasound diagnostic instrument

Philips 5500 Doppler ultrasonic diagnostic instrument in Holland (probe frequency is 2.5 MHz and 5.0 MHz) was used to measure left atrial dimension (LAD), Left ventricular end diastolic dimension (LVDd), Left ventricular end systolic dimension (LVDs), left ventricular posterior wall thickness (LVPWT), interventricular septal thickness (IVST), left ventricular ejection fraction (LVEF). The left ventricular myocardial weight (LVM) was calculated by Devereux formula, and the LVMI was calculated by body surface area (BSA). LVM(g) = 0.80 × 1.04 × [(LVDd + IVST + LVPWT)^3^ - LVDd^3^] + 0.60, BSA (m^2^) = 0.001 × Height (cm) + 0.0128 × body weight (kg) - 0.1529. The criteria for judging LVH are: LVMI > 115 g/m^2^ for men and LVMI > 110 g/m^2^ for women.

### Genetyping

Polymerase chain reaction (PCR) was performed in a final volume of 50 μl containing approximately 100 ng of DNA, 0.25 mMdNTPs,1.5 mM MgCl_2_, 100 ng of each primer, 1 × PCR buffer, and 0.02 U Taq DNA polymerase (Invitrogen Life Technologies, CA, USA). The amplification conditions for each PCR were 94°C for 5 min, followed by 30 cycles of 94°C for 30 s, 60°C for 50 s, and 72°C for 60s, with a post-cycling final extension of 5 min at 72°C. Primers used for the amplification of ApaI, BsmI, Taq I and Fok I were designed using Primer 6.0 (Table 1) [9]. The PCR products were verified using 1% agarose gel. Genotypes were confirmed by Sanger sequencing (Sangon, Shanghai, China).

**Table 1:** Primers used in the experiment

Polymorphisms	Forward primer(5’-3’)	Reverse primer (5’-3’)	Products (bp)
BsmI ( rs1544410)	CCTCACTGCCCTTAGCTCTG	TGCCTCCAAAATCAATCAGG	247
ApaI (rs7975232)	GGATCCTAAATGCACGGAGA	ACGTCTGCAGTGTGTTGGAC	265
TaqI (rs731236)	CAGAGCATGGACAGGGAGCAA	CACTTCGAGCACAAGGGGCGTTAGC	501
FokI (rs2228570)	AGCTGGCCCTGGCACTGACTCTGGCTCT	ATGGAAACACCTTGCTTCTTCTCCGTC	267

### Real-time polymerase chain reaction (RT-PCR)

Total RNA in cells was extracted using TRIzol reagent (Invitrogen Life Technologies, CA, USA). The concentration of RNA was detected using spectrophotometer, and reverse transcription was carried out by using TaKaRa reverse transcription kit (TaKaRa, Dalian, China) and T100 PCR instrument (Bio-Rad, USA). The qRT-PCR detection of cDNA obtained after reverse transcription was carried out by sybr-green (TaKaRa, Dalian, China) and fluorescence quantitative PCR detection system (Bio-Rad, USA). The expression of VDR mRNA was detected by qRT-PCR, with GAPDH as internal reference [10]. Primer sequences for VDR: 5’-GTGGACATCGGCATGATGAAG-3’ (forward) and 5’-GGTCGTAGGTCTTATGGTGGG-3’(reverse).

### Western blot

Total protein was extracted using RIPA buffer (Dingguo, Beijing, China), and the concentration of protein was determined by BCA method. Then 50 μg of protein sample was taken for loading, electrophoresis and membrane transfer. The obtained bands were blocked by 5% BSA for 1 min. Then rabbit anti-human GAPDH and VDR antibodies (Cell Signaling Technology, USA) (both diluted at 1:1 000) were added and incubated at 4°C overnight. PBST washed for 3 times × 5 min. Then corresponding secondary antibodies (diluted at 1:1 000) were added and incubated at 37°C for 1 h. After PBST washed for 3 times × 5 min, ECL chemiluminescence was used for band development [11].

### Statistical analysis

SPSS 17.0 software was used for statistical analysis. Hardy-Weinberg law of genetic balance was used to test genotype frequency. The measurement data were expressed by mean standard deviation, and the comparison between groups was performed by *t* test. X^2^ test was used for analysis of counting data. Pearson linear correlation analysis was used for analyzing the correlation between two variables. Taking multiple factors as covariates and VDR polymorphism as independent variables, the multivariate regression analysis was carried out by means of logistic equation. *p* < 0.05 was recognized as statistical significance.

## Results

### The main differences between MHD with LVH and without LVH

There were 120 maintenance hemodialysis patients, including 53 patients including 12 female with LVH and 67 patients including 20 female without LVH as control. All patients were in relatively stable condition, without other infection, inflammation, severe liver and other chronic diseases. The results showed that the risk of LVH in men was higher than that in women, and the risk of LVH also included prolonged dialysis time, increased heart rate, SBP, glaciated hemoglobin, iPTH, CRP concentration and decreased Calcium concentration (Table 2).

**Table 2:** Clinical differences between MHD patients with LVH and without LVH

MHD	With LVH(n = 53)	Without LVH(n = 67)	t/X^2^	*p*
Age (years)	52.7± 11.3	51.9 ± 12.5	1.98	0.112
BMI (kg/m^2^)	22.3 ± 3.20	23.1 ± 3.6	-1.869	0.063
Female, n (%)	12 (23%)	20 (30%)	-5.83	**0.031**
Dialysis time (y)	3.5 ± 2.4	2.1 ± 2.2	5.18	**< 0.001**
Heart rate (beats/minute)	90 ± 4.7	80 ± 2.3	4.43	**0.043**
SBP (mmHg)	146.4 ± 24.3	128.5 ± 24.1	-2.712	**0.017**
DBP (mmHg)	88.9 ± 13.7	87.7 ± 13.4	-0.535	0.391
Calcium (mmol/L)	1.9 ± 0.3	2.2 ± 0.2	-2.104	**0.036**
25(OH) D (ng/ml)	36.22 ± 2.88	37.54 ± 3.88	0.753	0.092
Phosphorus (mmol/L)	1.7 ± 0.6	1.8 ± 0.7	-0.861	0.235
iPTH (pg/mL)	446.9 ± 512.1	314.9 ± 369.2	2.487	**0.013**
Platelet(10^9^/L)	183.7 ± 61.9	170.7 ± 56.2	1.011	0.071
Hemoglobin (g/L)	116.8 ± 16.1	113.2 ± 14.9	0.987	0.101
Glycated hemoglobin(%)	6.1 ± 0.8	5.6 ± 0.6	3.819	**< 0.001**
Albumin (g/L)	35.8 ± 1.3	36.9 ± 3.1	-0.714	0.193
Triglycerides (mmol/L)	1.5 ± 1.1	1.6 ± 0.9	-0.923	0.316
Cholesterol (mmol/L)	3.5 ± 0.9	3.6 ± 0.9	-0.755	0.451
LDL (mmol/L)	1.9 ± 0.7	1.9 ± 0.7	-0.522	0.602
Urea nitrogen (mmol/L)	29.8 ± 8.4	30.3 ± 9.2	-1.243	0.101
Creatinine (mmol/L)	0.8 ± 0.3	0.7± 0.2	1.315	0.097
Beta microglobulin (μg/mL)	7.7 ± 5.2	7.4 ± 4.4	0.514	0.607
CRP (mg/L)	8.1 ± 4.4	6.8 ± 4.4	2.299	**0.022**

*SBP* systolic blood pressure; *DBP* diastole pressure; *BMI* body mass index; *LDL* low density lipoproteins; *iPTH* immunoreactive parathyroid hormone; *CRP* C protein

### Polymorphism of BsmI promotes the risk of LVH

Further study showed that Fok I (rs2228570), Bsm I (rs1544410), Apa I (rs7975232) and Taq I (rs731236) had two alleles and could be combined to form three genotypes. Among 120 MHD patients, bb genotype of Bsm I accounted for 79 cases. With the decrease of b genotype and increase of B genotype, LVH increased, and there was significant difference between MHD with LVH and without LVH (p = 0.01). Aa and AA are the main genotypes of Apa I, all three genotypes of FokI account for a certain proportion and TT is the main genotype of Taq I. However, Fok I, Apa I and Taq I polymorphisms had no significant difference between MHD with LVH and without LVH (Table 3). Further analysis showed that LAD, LVDd, LVDs, IVST and LVMI in BB + Bb genotype patients increased significantly (*p* < 0.05), as shown in Table 4.

**Table 3:** Distribution of VDR genotypes in MHD patients with LVH and without LVH

MDH	With LVH (n = 53)			Without LVH (n = 67)			*p*
Bsm I	BB	Bb	bb	BB	Bb	bb	0.01
	3	25	25	0	13	54	
Apa I	AA	Aa	aa	AA	Aa	aa	0.32
	27	23	3	31	29	7	
Fok I	FF	Ff	ff	FF	Ff	ff	0.38
	16	23	14	24	33	10	
Taq I	TT	Tt	tt	TT	Tt	tt	0.24
	50	3	0	63	4	0

**Table 4:** Correlation analysis between Bsm I alleles and biochemical index

MDH	bb (n = 79)	Bb (n = 38)	BB (n = 3)	*p*
Female, n (%)	21 (26.6%)	11 (28.9%)	0 (0)	0.47
Dialysis time (y)	2.9 ± 2.9	3.3 ± 3.1	2.8 ± 1.5	0.34
SBP (mmHg)	137 ± 25	143 ± 24	147 ± 22	0.06
Heart rate (b/min)	80 ± 10	80 ± 10	76 ± 9	0.31
Glycated hemoglobin(%)	5.8 ± 0.5	5.9 ± 0.7	6.1 ± 0.6	0.56
Calcium (mmol/L)	2.0 ± 0.93	2.1 ± 0.6	2.2 ± 0.9	0.93
iPTH (pg/mL)	312 ± 123.6	354.9 ± 223.9	366.9 ± 118.6	0.51
25(OH)D	36.13 ± 3.01	37.19 ± 2.34	38.05 ± 3.55	0.32
CRP (mg/L)	7.0 ± 4.4	7.6 ± 2.4	7.5 ± 3.4	0.44
Kt/V	1.3 ± 0.4	1.4 ± 0.2	1.4 ± 0.3	0.78
LVMI	118.1 ± 14.3	132.7 ± 14.5	136.9 ± 12.2	**0.004**
LAD	37.20 ±4.5	42.1 ± 5.6	42.2 ± 5.3	**0.013**
LVDd	46.3 ± 4.2	54.2 ±5.3	56.5 ± 5.9	**0.007**
LVDs	32.8 ±3.2	36.9 ± 3.1	37.1 ± 3.3	**0.02**
IVST	11.4 ± 0.9	13.5 ± 0.7	13.7 ± 0.6	**0.02**
LVPWT	12.4 ± 0.9	13.1 ± 0.7	12.8 ± 0.6	0.11

*LVMI* the left ventricular mass index; *LAD* left atrial dimension; *LVDd* left ventricular end diastolic dimension; *LVDs* left ventricular end systolic dimension; *LVPWT* left ventricular posterior wall thickness; *LVEF* left ventricular ejection fraction

Further logistic regression analysis between VDR polymorphism and biochemical index showed that Bsm I alleles are closely correlated with SBP, LVMI and LVDd; Apa I alleles are closely correlated with SBP and Taq I alleles are closely correlated with gender, SNP and Glycated hemoglobin (Table 5).

**Table 5:** Logistic regression analysis between biochemical index and VDR polymorphism

Variables	Bsm I alleles			Apa I alleles			Fok I alleles			Taq I alleles		
	OR	95% CI	*p*	OR	95% CI	*p*	OR	95% CI	*p*	OR	95% CI	*p*
Female, n (%)	1.341	1.544~3.176	0.185	1.882	1.768~3.844	0.109	1.053	1.553~2.474	0.194	3.345	1.058~2.943	**0.042**
Dialysis time (y)	2.134	1.568~3.117	0.231	2.655	1.265~2.486	0.146	1.746	0.973~2.032	0.155	2.765	1.423~2.356	0.178
SBP (mmHg)	5.105	1.512-2.033	**0.039**	4.347	1.087~1.732	**0.048**	2.765	0.945~2.038	0.065	3.934	1.037~2.134	**0.029**
Heart rate (b/min)	0.745	1.055~3.093	0.287	0.838	1.209~5.644	0.073	0.644	1.237~6.033	0.105	0.318	1.054~4.672	0.084
Glycated hemoglobin(%)	1.326	0.654~1.967	0.196	1.546	1.764~4.472	0.119	1.634	2.219~4.569	0.063	6.542	2.094~3.113	**0.018**
Calcium (mmol/L)	0.552	1.425~3.165	0.417	0.376	1.555~3.412	0.128	0.994	1.704~2.848	0.055	0.843	1.464~2.789	0.061
iPTH (pg/mL)	0.178	1.423~2.359	0.093	0.487	1.192~2.556	0.087	0.786	1.055~2.973	0.085	0.368	1.229~2.359	0.101
25(OH)D	1.092	0.757~1.904	0.056	1.655	0.532~2.845	0.162	2.973	0.902~1.869	0.072	2.148	0.691~1.886	0.067
CRP (mg/L)	0.843	1.232~5.986	0.156	2.437	1.174~5.491	0.144	2.388	0.935~4.743	0.189	1.065	1.044~6.388	0.202
Kt/V	1.555	2.213~4.568	0.563	3.883	1.967~4.855	0.348	4.176	1.692~3.455	0.253	4.157	1.856-3.255	0.088
LVMI	0.401	1.703~2.405	**0.019**	0.956	1.574~4.119	0.284	0.882	1.765~3.666	0.163	0.821	1.458~2.146	0.086
LAD	1.712	1.112~2.658	0.065	2.521	0.978~2.865	0.094	3.827	1.423~2.356	0.059	2.875	1.429~2.054	0.073
LVDd	1.513	1.235~2.415	**0.043**	3.144	0.713~1.676	0.104	0.774	0.757~1.904	0.162	3.189	0.554~2.578	0.091
LVDs	1.662	1.085~4.667	0.058	2.954	1.238~4.875	0.083	2.617	1.232~4.336	0.061	2.478	0.970~5.986	0.105
IVST	1.286	1.908~5.019	0.056	1.905	2.095~4.833	0.111	0.885	2.213~4.569	0.144	4.104	1.905~5.014	0.098
LVPWT	1.532	1.532~3.577	0.315	2.745	1.573~4.115	0.152	2.683	1.769~3.846	0.322	3.034	1.452~3.755	0.199

*OR* odds ratio; *CI* confidence intervals

### The expression of VDR in MHD patients with LVH was significantly down-regulated

**Fig. 1:**
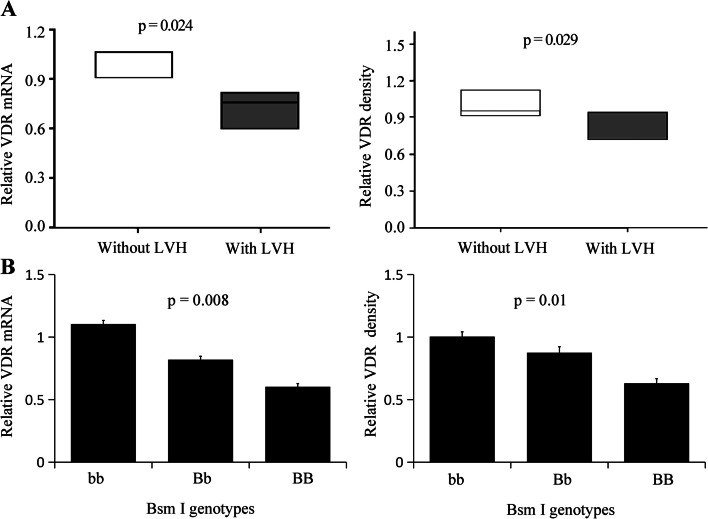
The expression of VDR (**A**) and relationship between BsmI polymorphism and VDR expression (**B**). Data are mean ± SD

Using real-time PCR and Western blot, we found that the VDR expression level decreased significantly in MHD patients with LVH (Fig. 1A), and correlation analysis showed that B allele was positively correlated with VDR mRNA (r = 0.66, *p* = 0.008) and VDR protein (r = 0.53, *p* = 0.01) (Fig. 1B).

## Discussion

Current studies show that changes in metabolites can cause changes in multiple system s[12]. Genotype differences are related to levels of metabolites in the body. The results of this study showed that VDR gene Bsm I had bb, Bb and BB three genotpes, and their genotype distribution accords with Hardy-Weinberg genetic balance. Studies on VDR gene polymorphism showed that Bsm I allele distribution vary greatly with different races [13]. The allele B of Bsm I is about 10% in China, while it reaches more than 35% in Caucasian population. The genotype ratio of bb is more than 90%, followed by Bb (less than 2%) in China, but Bb is more than 45% ~ 55% in Caucasian population, and BB is more than 18%. Among 120 MHD patients in the present study, there were 3 cases of BB type (2.5%), 38 cases of Bb type (31.7%) and 79 cases of bb type (65.8%).

Active vitamin D or VDR receptor agonist is helpful to improve atherosclerosis, cardiovascular calcification and myocardial hypertrophy. However, some reports showed that they might aggravate adverse reactions [14]. It is speculated that VDR gene polymorphism may play an important role in it. Ozdemir found that BB genotype of BsmI gene in Turkish dialysis patients was significantly higher than bb genotype, which was related to the severity of secondary hyperparathyroidism [15]. However, a Japanese study on gene polymorphism of Bsm I in 877 dialysis patients showed that serum PTH level of BB type patients was significantly lower than that of bb type patients, and b allele may be related to severe secondary hyperparathyroidism, while B allele is the protective gene of dialysis patients [16]. In this study, the B allele in MHD patients with LVH increased significantly. Correlation analysis showed that there was no significant correlation between the Bsm I genotypes and iPTH. However, compared with b allele patients, the related indexes of LVH in Bb and BB patients increased significantly, indicating that B allele may be susceptible to LVH risk in MHD patient. Moreover, we found that VDR gene polymorphism had significant correlations with VDR mRNA and protein, but no correlation with Calcium and 25(OH) D levels, indicating that VDR may play a role in the process of 25(OH) D function.

At present, the relationship between VDR gene polymorphism and LVH in MHD patients has been preliminarily studied. Testa found that LVMI of BB genotype patients increased significantly in BsmI gene polymorphism sites of MHD patients by following up 182 MHD patients for 2 years, and the occurrence of LVH was significantly related to B allele [17]. EI-Shehaby also found that although the distribution of Bsm I genotype in Egyptian dialysis patients is roughly consistent with that of healthy people, the frequency of B allele is obviously increased, and it is significantly correlated with low active vitamin D level, high PTH and LVH [18]. In this study, no significant differences in blood calcium, phosphorus, creatinine, blood pressure and KT/V value among different genotypes. It is speculated that the traditional mechanisms affecting LVH can be positively corrected by controlling hypertension, using erythropoietin to correct anemia, fully dialysis to remove uremic toxin, proper ultrafiltration, phosphorus restriction and discharge and taking active vitamin D, but patients with B allele had significant correlation with LVH when compared b allele.

Rubello studied 75 patients after renal transplantation, and found that VDR gene polymorphism can change the VDR function on the surface of parathyroid gland, and make the inhibition of active vitamin D on the transcription of pre-pro-PTH gene mRNA in parathyroid cells weaken, and increase the translation and synthesis of PTH [19]. High PTH not only easily leads to renal osteodystrophy, but also can cause extensive calcification and LVH in cardiovascular system, which is an important factor aggravating the death of dialysis patients. It is speculated that the genetic polymorphism of Bsm I site may directly change the sensitivity of VDR or its expression in target organs. In the present study, we found the VDR mRNA and protein expression decreased in B allele patients.

Although we found no correlation between the Apa I, Fok I or Taq I polymorphism and LVH, there were significant correlations between VDR polymorphism and other risk factors of cardiovascular diseases, which may have an indirect effect on LVH formation. The relevant literature shows that vitamin D deficiency and Taq-I polymorphism are associated with stage 2 hypertension, depending on age and BMI, in postmenopausal women [20]. Moreover, improvements in metabolic profile due to vitamin D supplementation is influenced by VDR polymorphisms, specifically for carriers of Taq-I GG and Bsm-I BB genotypes [21]. Another study shows that Taq-I polymorphism leads to an increase in the risk of ischemic stroke in a gender specific manner through impaired lipid [22]. Our results also showed that Apa I alleles are closely correlated with SBP and Taq I alleles are closely correlated with gender, SBP and Glycated hemoglobin, indicating VDR polymorphism gets involved in the body’s metabolism and SBP, and may indirectly affect heart function.

Fibroblast growth factor-23 (FGF-23) is an established inducer of LVH in renal patients. Moe reported that treatment with calcimimetic cinacalcet significantly lowers serum FGF23, which are associated with lower rates of cardiovascular death and major cardiovascular events [23]. Wolf reported that etelcalcetide potently lowers FGF23 in patients with secondary hyperparathyroidism receiving hemodialysis and that the effect remains detectable among patients who receive concomitant treatments aimed at mitigating treatment-associated decreases in serum calcium [24]. Dörr found that FGF23 suppression by etelcalcetide inhibited the progression of LVH compared with alfacalcidol in hemodialysis patients [25]. New extended-release calcifediol deserved lesser impact on FGF-23 levels [26]. FGF-23 was not tested in this study. Whether VDR polymorphism affects the expression of FGF-23 requires further study. Besides, Vitamin D binding protein (VDBP) is the primary vitamin D carrier and many of its genetic polymorphisms are able to induce the expression of proteins with different affinities for the vitamin D, which in turn might affect its serum levels and CAD incidence. However, Although VDBP polymorphisms can induce the severity of infection [27, 28], there is no strong evidence of an impact on CVD, because VDBP level is associated neither with disease outcome nor with vitamin D status. The GC gene variant also has no effect on 25(OH) D levels [29–32].

## Conclusion

The results showed that VDR Bsm I gene polymorphism played an important role in estimating LVH risk of MDH patients, and provided theoretical basis for early detection and active prevention of cardiovascular complications and improvement of prognosis of patients with end-stage renal disease.

## Data Availability

The datasets generated and/or analysed during the current study are not publicly available due to limitations of ethical approval involving the patient data and anonymity but are available from the corresponding author on reasonable request.

## Abbreviations

SNP: Single nucleotide polymorphisms;
VDR: Vitamin D receptor
LVH: Left ventricular hypertrophy
MHD: Maintenance hemodialysis
CVD: Cardiovascular disease
LVMI: Left ventricular mass index
